# Pyogenic liver abscess after laparoscopic sleeve gastrectomy

**DOI:** 10.12669/pjms.343.14409

**Published:** 2018

**Authors:** Hakan Demir, Kayhan Ozdemir, Kerem Karaman

**Affiliations:** 1 Dr. Hakan Demir, Department of General Surgery, Sakarya University Teaching and Research Hospital, Sakarya, Turkey; 2 Dr. Kayhan Ozdemir, Department of General Surgery, Sakarya University Teaching and Research Hospital, Sakarya, Turkey; 3 Dr. Kerem Karaman, Associate Professor, Department of General Surgery, Sakarya University Teaching and Research Hospital, Sakarya, Turkey

**Keywords:** Pyogenic liver abscess, Sleeve gastrectomy, Staple line leak

## Abstract

An infected material in the gastrosplenic area after laparoscopic sleeve gastrectomy (LSG) due to hematoma or staple line leak has the potential to spread of the bacterial content to the liver which can result in pyogenic liver abscess. Presently described is a thirty-seven-year-old female patient with unilocular pyogenic liver abscess two weeks after LSG. The abscess resolved by Ultrasound guided percutaneous drainage plus intravenous antibiotic treatment. Review of the literature regarding 3 other cases with liver abscess after LSG is also presented.

## INTRODUCTION

Laparoscopic sleeve gastrectomy (LSG) is performed worldwide with increasing frequency in morbidly obese patients.^1^ Reasons to prefer LSG over other bariatric procedures is its easy technique which does not need anastomosis; preserving of the pylorus; avoidance of complications associated with malabsorptive operations such as dumping syndrome and diarrhea; and less need for trace elements and vitamin supplements. On the other hand, LSG has the longest staple line which increases the risk of staple line bleeding and staple line dehiscence.^2^

Hemorrhage during or after SG is a rare complication, which can sometimes be problematic in that it may trigger a series of events such as hematoma, followed by abscess formation, which will require percutaneous drainage or re-operation. Further, an infected hematoma around the staple line has the potential risk to develop leaks.^3^,^4^ We report a rare complication of a case that had developed pyogenic liver abscess two weeks after LSG.

## CASE REPORT

**Fig.1:**
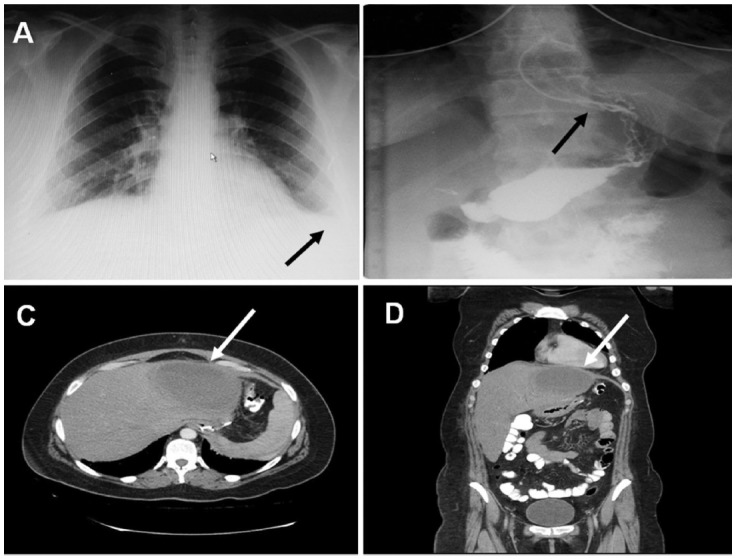
A) Chest X-ray showed blunting of the left costaphrenic angle with minimal left pleural effusion, B) oral contrast given gastro duodenography which ruled out staple line leak, C) Axial section of the liver abscess, D) Vertical image of the liver abscess.

A thirty-seven-year-old female patient presented with abdominal pain and fever. Two weeks ago, she underwent LSG in a state hospital. Physical examination revealed minimal sensitivity by palpation localized to the left upper quadrant. Her body temperature was 39°C. White blood cell count was 11,600mm^3^/dL, and the C-reactive protein (CRP) value was 166. The chest X-ray showed blunting of the left costa-phrenic angle with minimal left sided pleural effusion (Fig.1-A). An oral contrast given esophago-dueodenography was performed to rule out a staple line leak under scope which did not show extra-luminal contrast extravasations (Fig.1-B). Abdominal computerized tomography (CT) revealed a unilocular pyogenic liver abscess measured 12x7cm in diameter which was localized to the left lobe (Fig.1-C, D). A history of LSG from two weeks ago and a left sided pleural effusion on chest X-Ray were suggestive of pyogenic liver abscess secondary to staple line leak. The patient was hospitalized and an Ultrasound guided percutaneous drainage catheter was placed. Intravenous antibiotic therapy (piperacillin-tazobactam3x4.5gr/day) was started immediately. The course of the patient was uneventful. The liver abscess resolved and she was discharged on the 22^th^ day with oral antibiotic therapy.

## DISCUSSION

Bleeding from the staple line during and after LSG represents the most frequent complication at a rate of up to 9.3% (mean rate 3.6%).^5^ Gastric staple line leak is another mortal complication. It requires prompt and meticulous handle such as percutaneous drainage for converting the leak material to a controlled fistula and / or endoscopically intragastric covered stent placement. Both infected hematoma or abscess material around the staple line are strong risk factors for staple line dehiscence. However, formation of abscess in this area may also develop during the division of the gastric fundus from the short gastric arteries which can leads to partial ischemia of the spleen with subsequent infarction and necrosis. It is also possible to think the opposite where an asymptomatic minor leak from the staple line triggers an isolated splenic abscess due to bacterial seeding. There are few case reports in the literature regarding the formation of splenic abscess after LSG.^6^,^7^

Staple line leaks are classified according to time of occurrence, clinical symptoms (fever, tachycardia, abdominal pain and sepsis) localization of the leak (fundus or antra-pyloric region); and radiological signs. Early staple line leaks occur in the first four postoperative days usually due technical flaws such as improper fire of the stapler and / or incorrect cartridge selection. Intermediate staple line leaks occur between the 4^th^ and 9^th^ postoperative days which are mostly related with infection or ischemic insults of the gastric fundus. Late gastric leaks (≥ 9 days) occur with high probability due to abscess formation around the staple line.^4^ In the presence of staple line leak; symptoms and signs usually accompany such as fever, abdominal pain, tachypnea, tachycardia and leukocytosis. Left pleural effusion on chest X-ray and extra-luminal contrast extravasation by computerized tomography are strong radiological signs associated with staple line leak. However, although rare, clinical symptoms and signs may remain obscure. In case of doubt, contrast enhanced abdominal computerized tomography is the most sensitive diagnostic method for screening staple line leak, hematoma and abscess formation.^8^

An infected material in the gastrosplenic area due to hematoma or staple line leak has the potential to spread of the bacterial content to the liver which can result in pyogenic liver abscess. Although this probability is extremely rare, there are previously reported 3 cases with liver abscess after LSG, and the present case is the fourth.^7^,^9^

In the present case, it has been thought that, an asymptomatic minor leak at the staple line resulted in migration of the bacteria to the liver where the leak at the staple line during this process spontaneously healed. Blunting of the left costa-phrenic angle with minimal left sided pleural effusion on chest X-Ray during her admission strongly suggested evidence of a migrated staple line leak.

Another possible risk factor for the development of liver abscess after LSG is ascending migration of bacteria from the portal venous system due to pylephlebitis of the porto-mesenteric veins secondary to leak-related collection.^10^ However, pylephlebitis was not detected in CT images of the present case.

Management of liver abscesses after LSG should be considered according to degree and extends of the disease. In the absence of sepsis of a hemodynamically stable patient with an isolated pyogenic liver abscess, percutaneous drainage with intravenous antibiotic treatment is usually sufficient. On the other hand, surgical intervention may be required when peritonitis signs accompany to liver abscess. In this situation, staple line leak should be ruled out by oral contrast given abdominal computerized tomography. In the presence of staple line leak, endoscopic interventions including covered stent placement or clips usage are options to close the defective area. A drainage catheter placement whether by image-guided percutaneous route or surgically is necessary to convert the gastric leakage content to a controlled fistula.^7^,^8^ In the present case, because of an isolated unilocular pyogenic liver abscess without a staple line leak, percutaneous drainage catheter placement with antibiotic therapy was sufficient.

## CONCLUSION

In conclusion, development of pyogenic liver abscess after LSG is an extremely rare clinical entity. Image-guided percutaneous drainage plus intravenous antibiotic therapy is usually sufficient in a hemodynamically stable patient with unilocular pyogenic liver abscess.

### Author`s Contribution

**HD** conceived, designed & editing of manuscript.

**KO** did data collection and manuscript writing.

**KK** did review and final approval of manuscript.
